# CXCL10 promotes melanoma angiogenesis and tumor growth

**DOI:** 10.1080/19768354.2024.2402024

**Published:** 2024-09-11

**Authors:** Bongjun Kim, Yun-Yong Park, Jong-Ho Lee

**Affiliations:** a Department of Translational Molecular Pathology, The University of Texas MD Anderson Cancer Center, Houston, TX, USA; b Department of life Science, Chung-Ang University, Seoul, Republic of Korea; c Department of Health Sciences, The Graduate School of Dong-A University, Busan, Republic of Korea; d Department of Biomedical Sciences, Dong-A University, Busan, Republic of Korea

**Keywords:** CXCL10, melanoma, angiogenesis, tumor growth, pro-angiogenic factors

## Abstract

Upregulation of CXC motif chemokine 10 (CXCL10) in melanoma patients has been found to be associated with melanoma progression. However, the role of endogenous CXCL10 from the host in melanoma tumor growth remains unclear. In the present study, we found that host-derived endogenous CXCL10 production was dramatically augmented during subcutaneous B16F10 melanoma tumor growth and that host ablation of CXCL10 in *Cxcl10^-/-^* mice showed a decrease in both angiogenesis and tumor growth of B16F10 melanoma *in vivo*. Several signaling pathways involved in production of pro-angiogenic factors and tumor growth were activated by CXCL10 in B16F10 melanoma cells. CXCL10 increased expression of pro-angiogenic factors, such as vascular endothelial growth factor (VEGF), platelet-derived growth factor subunit-B (PDGF-B), fibroblast growth factor 2 (FGF2), hepatocyte growth factor (HGF), and angiopoietin 2 (Angpt2), in B16F10 melanoma cells, resulting in enhanced tube formation and proliferation of human umbilical vein endothelial cells *in vitro*. In addition, CXCL10 directly enhanced B16F10 melanoma tumor growth in an *in vitro* three-dimensional cell culture system. Together, our findings reveal that amplified host-derived endogenous CXCL10 is critical for B16F10 melanoma angiogenesis and tumor growth. Therefore, CXCL10 might represent a therapeutic target for melanoma.

## Introduction

Angiogenesis, the formation of new blood vessels from existing capillaries, is a complex but tightly regulated process that eventually creates a complete, regular, and mature vascular network indispensable for both physiologic and pathologic processes (Hanahan and Weinberg 2011; Apte et al. 2019). It involves activation, proliferation, survival, and migration of endothelial cells regulated by various pro-angiogenic and/or anti-angiogenic factors (Apte et al. 2019). Angiogenesis is hyperactivated by the over-expression of pro-angiogenic factors and the inactivation of anti-angiogenic factors in the microenvironment of several malignant tumors (Khosravi Shahi et al. 2009; Hall et al. 2015; Zimna and Kurpisz 2015; Pandita et al. 2021). Tumor growth and metastasis highly rely on angiogenesis as the development of new blood vessels is crucial for continued growth and malignant dissemination of solid tumors (Hanahan and Weinberg 2011; Apte et al. 2019). Consequently, excessive angiogenesis is often observed in the pathogenesis of most solid tumors.

Melanoma is one of the most aggressive human cancers, responsible for over 75% of all skin cancer-related mortalities (Rebecca et al. 2020; Centeno et al. 2023). The incidence of melanoma has increased significantly over the past few decades (Li Z et al. 2022). The aggressiveness of melanoma is typically associated with oncogenic mutations and dysregulated expression of cancer related genes that can cause malignant transformation of melanocytes, cells derived from neural crest stem cells (Davies et al. 2002; Rebecca et al. 2020; Diener and Sommer 2021). Cutaneous melanoma is the most prevalent type. It is surgically curable in early stages with 5-year relative survival rates up to 98%. However, its 5-year survival rate drops to 23% in patients with a vertical growth phase phenotype that is highly invasive and metastasis (Rebecca et al. 2020). Angiogenesis has been shown to play an essential role in melanoma progression accompanied with vertical growth phase and metastatic growth (Cho et al. 2019; Pandita et al. 2021). Multiple pro-angiogenic factors, including vascular endothelial growth factor (VEGF) (Rofstad and Danielsen 1998; Rofstad and Halsor 2000), platelet-derived growth factor subunit-B (PDGF-B) (Barnhill et al. 1996; Rofstad and Halsor 2000), fibroblast growth factor 2 (FGF2) (Reed et al. 1994; Miglarese et al. 1997; Rofstad and Halsor 2000), hepatocyte growth factor (HGF) (Lezcano et al. 2014), and angiopoietin 2 (Angpt2) (Helfrich et al. 2009; Abdul Pari et al. 2020), are produced by primary melanoma cells to promote angiogenesis in an advanced melanoma tumor (Jour et al. 2016; Liu et al. 2023). Various lines of evidence have shown that an increase of angiogenesis, as evidenced by intratumoral microvessel density, is correlated with clinicopathological parameters (tumor thickness, overall survival, and relapse rate) in human melanoma patients (Srivastava et al. 1988, 1989; Fallowfield and Cook 1991; Kashani-Sabet et al. 2002). Therefore, angiogenesis serves as a significant marker of tumor aggressiveness and unfavorable clinical prognosis in human melanoma. The identification of angiogenic regulator in angiogenesis-induced vertical tumor growth will provide alternative therapeutic targets against melanoma.

CXC motif ligand 10 (CXCL10), also known as interferon gamma-induced protein-10, is a member of the subfamily in interferon-γ (IFN-γ) – inducible chemokines. It binds to its receptor CXCR3 to exert its well-characterized biological processes, such as leukocyte trafficking, adaptive immunity, and inflammation (Khan et al. 2000; Liu M et al. 2011). CXCL10 and its corresponding receptor CXCR3 are highly expressed by various cell types, including leukocytes and macrophages as well as some epithelial and cancer cells in a wide range of human disorders (Luster and Ravetch 1987; Garcia-Lopez et al. 2001; Kawada et al. 2004; Dyer et al. 2009; Lo et al. 2010; Altara et al. 2016; Bagheri et al. 2020). In inflamed tissues, CXCL10 recruits T-helper 1 (Th1) cells and upregulates IFN-γ production, which in turn stimulates CXCL10 expression in various cell types, resulting in a positive feedback for Th1 responses and CXCL10 amplification (Rotondi et al. 2007). In particular, overexpression of CXCL10 and CXCR3 has been associated with advanced human cancers, including malignant melanoma. It has been correlated with a poor prognosis in melanoma patients (Monteagudo et al. 2007; Jiang et al. 2015; Wightman et al. 2015; Bagheri et al. 2020). Recently, Wightman *et al.* have reported that autocrine CXCL10/CXCR3 axis is critical in enhancing melanoma metastasis to the lung and metastatic recurrence (Wightman et al. 2015). In line with this, we have previously reported that endogenous CXCL10 can facilitate trafficking of CXCR3-expressing melanoma cells to bone, in which direct interactions between melanoma cells and macrophages can further stimulate CXCL10 amplification from macrophages to promote melanoma metastatic growth and subsequent osteolysis (Lee JH et al. 2012). However, the role of host-derived endogenous CXCL10 in melanoma angiogenesis and vertical growth remains elusive.

In this study, using B16F10 murine melanoma tumor cells with *Cxcl10^-/-^* syngeneic C57BL/6 mice, we demonstrated that host-derived endogenous CXCL10 was amplified during subcutaneous B16F10 melanoma tumor growth. Such amplification was indispensable for melanoma angiogenesis and primary tumor growth *in vivo*. We found that CXCL10 activated signaling pathways involved in pro-angiogenic factor expression and tumor growth in B16F10 melanoma cells. CXCL10 induced expression of pro-angiogenic factors in B16F10 melanoma cells and subsequent tube formation of human umbilical vein endothelial cells (HUVECs) *in vitro*. In addition, CXCL10 directly enhanced B16F10 melanoma tumor growth in an *in vitro* three-dimensional cell culture system.

## Materials and methods

### Materials

Rabbit polyclonal antibodies that could recognize phospho-ERK (Thr202/Tyr204; #9101; 1:1,000 for immunoblotting), ERK (#9102; 1:1,000 for immunoblotting), phospho-AKT (Ser473; #4060; 1:2,000 for immunoblotting), AKT (#9272; 1:1,000 for immunoblotting), phospho-JNK (Thr183/Tyr185; #9251; 1:1,000 for immunoblotting), JNK (#9252; 1:1,000 for immunoblotting), phospho-Jak2 (Tyr1007/1008; #3771, 1:1,000 for immunoblotting), Jak2 (#3230; 1:1,000 for immunoblotting), phospho-CREB (Ser133; #9198, 1:1,000 for immunoblotting), and CREB (#4820; 1:1,000 for immunoblotting) were purchased from Cell Signaling Technology (Danvers, MA, USA). Rabbit polyclonal antibody for CD31 (ab28364; 1:300 for immunohistochemistry) was purchased from Abcam (Cambridge, MA, USA). Recombinant mouse CXCL10 (#250-16) and human CXCL10 (#300-12), TGF-β (#100-21C), and IL-1 (#200-01A) were obtained from PeproTech (London, UK).

### Cells

B16F10 murine melanoma cells, 4T1 murine breast carcinoma cells, MDA-MB-231 human breast carcinoma cells, and PC-3 human prostate carcinoma cells were purchased from the Korean Cell Line Bank (KCLB; Seoul, Republic of Korea) and maintained in Dulbecco’s modified Eagle’s medium (DMEM; #LM001-05, Welgene, Korea) supplemented with 10% fetal bovine serum (#S001-04, Welgene, Gyeongsan-si, Korea) and 1% Penicillin/Streptomycin (#PS-B, Capricorn Scientific, Hessen, Germany). All cells were routinely tested for mycoplasma with negative results. B16-FL cells were generated from B16F10 cells by stable transfection of the firefly luciferase gene.

### Quantitative real-time PCR analysis

Total RNA isolation, reverse transcription (RT), and real-time PCR were conducted as described previously (Yun et al. 2023). All reactions were run in triplicate and normalized to the housekeeping gene *β-actin*. The following primer pairs were used for quantitative real-time PCR: mouse *vegf a*, 5′-TGCAGATTATGCGGATCAAACC-3′ (forward) and 5′-TGCATTCACATTTGTTGTGCTGTAG-3′ (reverse); mouse *pdgf-b*, 5′-GATCTCTCGGAACCTCATCG-3′ (forward) and 5′-GGCTTCTTTCGCACAATCTC-3′ (reverse); mouse *fgf2*, 5′-AGCGGCTCTACTGCAAGAAC-3′ (forward) and 5′-GCCGTCCATCTTCCTTCATA-3′ (reverse); mouse *hgf*, 5′-ACTGACCCAAACATCCGAGTTG-3′ (forward) and 5′-TTCCCATTGCCACGATAACAA-3′ (reverse); mouse *angpt2*, 5′-TGCGGAAATCTTCAAGTCAGG-3′ (forward) and 5′-CCTTGATCTCCTCTGTGGAGTTG-3′ (reverse); mouse *β-actin*, 5′-ATGTGGATCAGCAAGCAGGA-3′ (forward) and 5′-AAGGGTGTAAAACGCAGCTC-3′ (reverse).

### Immunoblot analysis

Immunoblot analysis was performed as previously described (Lee TW and Lee 2022; Son et al. 2022) with some modifications. Briefly, proteins were extracted from cultured cells using a cell lysis buffer (50 mM Tris-HCl, [pH 7.5], 0.1% SDS, 1% Triton X-100, 150 mM NaCl, 1 mM DTT, 0.5 mM EDTA, 100 µM sodium orthovanadate, 100 µM sodium pyrophosphate, 1 mM sodium fluoride, and proteinase inhibitor cocktail). Cell extracts were centrifuged at 15,000 rpm for 15 min at 4℃. Protein concentrations of cell lysates were determined using the DC protein assay Kit (#5000112, Bio-Rad, Hercules, CA, USA). Equal amounts of proteins in cell lysates were resolved by SDS-PAGE. Proteins were then transferred to a nitrocellulose membrane. The membrane was blocked with 5% skim milk in TBST (Tris-buffered saline with 0.1% Tween® 20 Detergent) at room temperature for 30 min and then incubated with indicated antibodies at 4℃ overnight. The membrane was then incubated with horseradish peroxidase-conjugated secondary antibodies (anti-rabbit, #NA934V, Sigma Aldrich, St. Louis, MO, USA or anti-mouse, #NA931V, Sigma Aldrich, MO, USA) at room temperature for 2 h. Band intensity was quantified using ImageJ 1.53e software (National Institutes of Health). Each experiment was repeated at least three times.

### Tube formation assay

Human umbilical vein endothelial cells (HUVECs) were maintained in Endothelial Cell Growth Basal Medium-2 (EBM-2; #CC-3156, Lonza, Walkersville, MD, USA) supplemented with SingleQuots^TM^ Supplements and Growth Factors (#CC-4176; Lonza, Walkersville, MD, USA). Matrigel (#354234; Corning, Flintshire, UK) was diluted with serum-free EBM-2 medium and used to coat 96-well plates at 37℃ for 1 h. HUVECs were seeded into a 96-well plate in EBM-2 or conditional medium at a density of 1 × 10^4^ cells per well. After 4 h, the number of tube formation was imaged and analyzed using phase contrast microscopy and ImageJ software version 1.53e (National Institutes of Health).

### Cell proliferation assay

HUVECs were seeded at a density of 1 × 10^3^ cells/well in a 96-well plate and cultured under the indicated experimental conditions. Cell proliferation was measured using the Quanti-Max WST-8 cell viability assay kit (#QM2500; BIOMAX, Korea) according to the manufacturer’s instructions.

### Preparation of conditioned medium

B16F10 cells were cultured with DMEM complete medium in 10-cm tissue culture dishes until optimal confluence (70%) and then treated with or without recombinant mouse CXCL10 (100 ng/ml) for 24 h. These cells were washed three times with serum-free DMEM and then cultured in fresh EBM-2 media. Conditioned medium (CM) was harvested after 24 h of incubation, centrifuged at 2,000 rpm for 5 min, and stored at – 80℃.

### 3D on-top cell culture

3D on-top culture was performed as previously described (Lee GY et al. 2007) with some modifications. Briefly, prechilled 24-well culture plates were coated with a thin layer of Engelbreth-Holm-Swarm (EHS) tumor extract (Matrigel; #354234, Corning, Flintshire, UK) and incubated at 37°C for 30 min. Subsequently, a cell suspension (0.5 × 10^3^ cells in 500 µl of complete medium containing 2% EHS with or without CXCL10 at 100 ng/ml) was added on top of coated EHS layer. The medium was changed every 3–4 days and the culture was maintained for 10 days. Cells were maintained in a humidified atmosphere incubator (5% CO_2_) at 37°C. The size and number of colonies were analyzed using an osteomeasure image analysis system (Osteo-Metrics, Decatur, GA, USA).

### Animal studies

*Cxcl10^-/-^* mice (C57BL/6 background) were obtained from The Jackson Laboratory (Bar Harbor, ME, USA). After 5 × 10^5^ B16-FL cells were subcutaneously injected into 5-week-old male C56BL/6 mice (5 mice/group), tumor growths in wild-type (WT) and *Cxcl10^-/-^* mice were analyzed using the bioluminescence imaging at days 5, 10, and 21. Mice were sacrificed and their sera were collected at 21 days. These mice then underwent blinded necropsy. All animal procedures were reviewed and approved by the Dong-A University Institutional Animal Care and Use Committee.

### ELISA

Serum protein levels of mouse CXCL10 were measured using a Mouse CXCL10 DuoSet ELISA kit (#DY466; R&D Systems, Minneapolis, MN, USA) according to the manufacturer’s instructions.

### Bioluminescence imaging and analysis

Mice were injected intraperitoneally with 150 mg/kg D-luciferin (XENOGEN) in PBS 10 min before imaging. Imaging was performed using a charge-coupled device camera (IVIS 100; exposure time of 1 or 3 min, binning of 8, field of view of 15 cm, f/stop of 1, and no filter). Mice were anesthetized by isoflurane (2% vaporized in O_2_) and shaved to minimize attenuation of signal by pigmented black hair. For analysis, total photon flux (photons per second) was measured from a fixed region of interest using Living Image software. Bioluminescent signals within the fixed region of interest were normalized to the background luminescence and obtained over the same region of interest from animals that had not been injected with D-luciferin.

### Histological and immunohistochemical analyses

Solid tumors were removed, fixed with 4% paraformaldehyde, and embedded in paraffin. Histological sections (5μm in thickness) were prepared and stained with hematoxylin and eosin (H&E) or subjected to immunohistochemical staining for CD31, as described previously (Lee JH et al. 2012; Lim et al. 2022). Nuclei were stained with hematoxylin. Six randomly chosen fields per slide were analyzed and averaged.

### Genomic data analysis

Genomic datasets were downloaded from The Gene Expression Omnibus (GEO) database (https://www.ncbi.nlm.nih.gov/geo) and cBioPortal (https://www.cbioportal.org) database. They were then processed using R-package. Correlation analysis between two genes was performed with *Pearson*’s correlation analysis. *P*-value indicates the significance of correlation.

### Statistical analysis

All quantitative data are presented as mean ± SD of at least three independent experiments. The normality of data was assessed using Shapiro–Wilk test. Means of two groups were compared using Student’s t-test. Normality and homogeneity assumptions were met. The SPSS statistical package version 12 (SPSS Inc., Chicago, IL, USA) was used for all statistical analyses. Values of *P* < 0.05 indicated statistically significant differences.

## Results

### Host-deficiency of CXCL10 decreases melanoma tumor growth and angiogenesis *in vivo*

**Figure 1.**
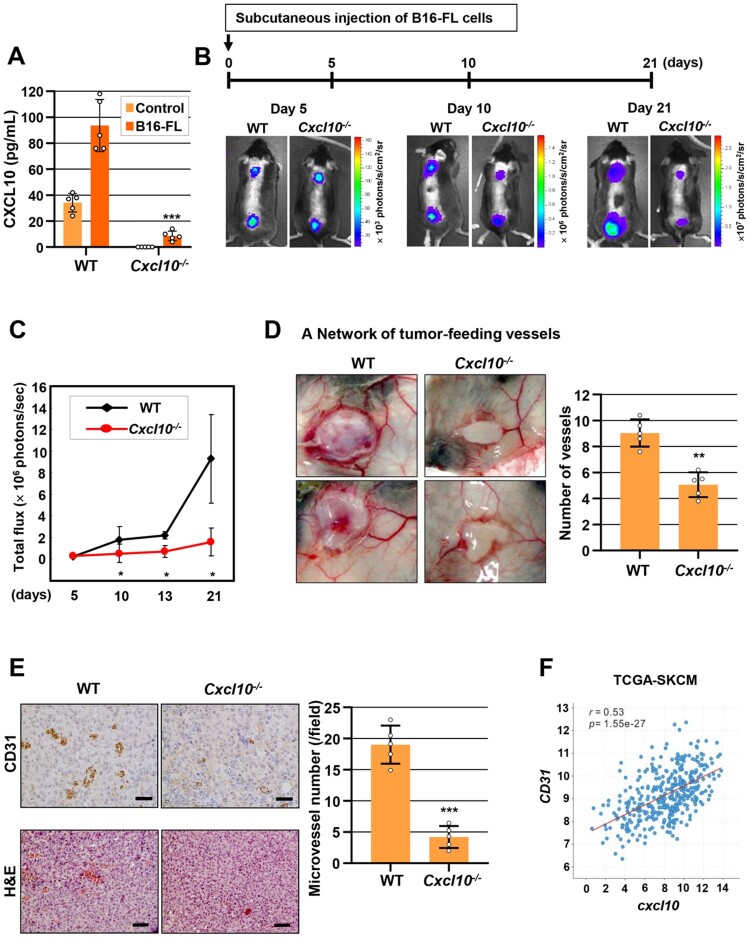
**Host-deficiency of CXCL10 decreases melanoma tumor growth and angiogenesis *in vivo.*** B16-FL cells were subcutaneously injected into WT and *Cxcl10^-/-^* mice. Tumor burden and tumor angiogenesis were then analyzed. **(A)** Serum CXCL10 levels at day 21 in non-tumor-bearing or B16-FL tumor-bearing WT and *Cxcl10^-/-^* mice. **(B)** A schematic diagram of experimental procedures (upper panel). Representative bioluminescence images on days 5, 10, and 21 after injection of B16-FL cells (bottom panel). **(C)** Bioluminescence imaging analysis of tumor burden on indicated days. **(D)** Gross of tumor-feeding vessels at 10 days after tumor formation. Quantification of tumor-feeding vessels is shown. **(E)** IHC analyses of tumor tissues with an anti-CD31 antibody (upper images) and H&E staining (bottom images) at 5 days after tumor formation (left panel). Quantification of microvessel number (right panel) is shown. Scale bar, 50 μm (CD31) and 100 μm (H&E). **(F)** TCGA correlation analyses between *Cxcl10* mRNA expression and *CD31* mRNA expression from TCGA-SKCM (skin cutaneous melanoma) data (*n *= 468). Data are presented as mean ± SD of three independent experiments (**A, C, D, E**). *, P < 0.05; **, *P *< 0.01; ***, *P *< 0.001, based on the Student’s t-test.

To investigate whether host-expressing CXC10 is essential for melanoma tumor growth, B16-FL cells (B16F10 murine melanoma cells stably expressing firefly luciferase) were subcutaneously implanted into wild-type (WT) and *Cxcl10^-/-^* syngeneic C57BL/6 mice. Consistent with our previous results that CXCL10 production was increased during bone colonization of B16F10 (Lee JH et al. 2012), the serum CXCL10 levels in WT mice were markedly elevated by subcutaneous injection of B16-FL cells (Figure 1(A), left bars), whereas only marginal increase was observed in *Cxcl10^-/-^* mice (Figure 1(A), right bars). We next determined tumor growth by monitoring with bioluminescence imaging (BLI), which showed that tumor burden was significantly decreased in *Cxcl10^-/-^* mice than in WT mice (Figure 1(B and C)). Interestingly, when we exposed an intact subcutaneous tumor at autopsy, we found that tumor growth suppression was correlated with angiogenesis inhibition. As shown in Figure 1(D), melanoma tumors in WT contained several blood vessels inside the tumor as well as blood vessels directly feeding into the tumor visible on the underside of the skin, whereas tumors in *Cxcl10^-/-^* had few blood vessels in both inside tumor and tumor-feeding vessels directly on the tumor surface. Immunohistochemical analysis further revealed that microvessel density, as evidenced by the intensity of CD31 expression, was considerably decreased in *Cxcl10^-/-^* mice than in WT mice (Figure 1(E)), event at an early stage (day 5) in which tumor sizes were comparable between WT and *Cxcl10^-/-^* mice. To determine the clinical significance of CXCL10-induced angiogenesis, we performed genomic analyses from The Cancer Genome Atlas (TCGA) database. As shown in Figure 1(F), *Cxcl10* mRNA expression levels were positively correlated with expression levels of *CD31* mRNA in cutaneous melanoma. These results indicate that the host-derived endogenous CXCL10 expression is amplified, which is required for melanoma tumor growth and angiogenesis.

### CXCL10 induces expression of pro-angiogenic factors in B16F10 melanoma cells and enhances B16F10-mediated angiogenesis *in vitro*

**Figure 2.**
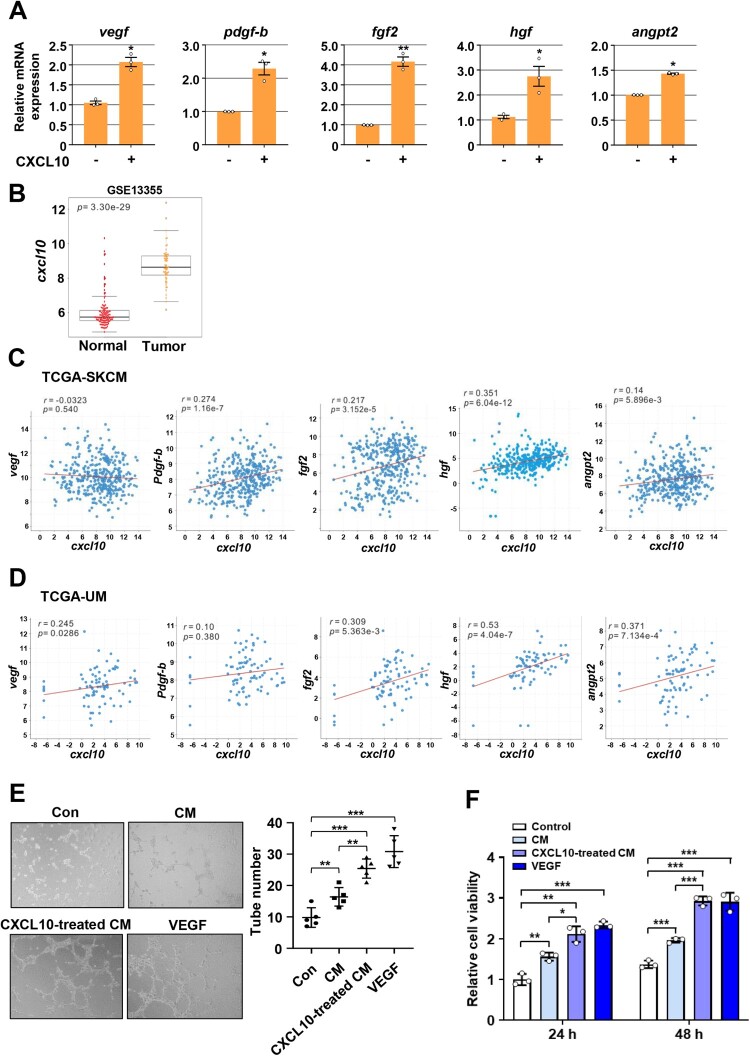
**CXCL10 induces expression of pro-angiogenic factors in B16F10 melanoma cells and enhances B16F10-mediated angiogenesis *in vitro***. (A) Relative mRNA expression levels of *vegf*, *pdgf-b*, *fgf2*, *hgf,* and *angpt2* in B16F10 cells treated with or without CXCL10 (100 ng/ml) for 24 h. **(B)** GEO analysis of *Cxcl10* mRNA expression in normal (*n* = 122) and melanoma tumor (*n* = 58) tissues from GSE13355 data. **(C** and **D)** TCGA correlation analyses between *Cxcl10* mRNA expression and *vegf*, *pdgf-b*, *fgf2*, *hgf,* or *angpt2* mRNA expression from TCGA-SKCM (skin cutaneous melanoma) data (C; *n *= 468) and TCGA-UM (uveal melanoma) data (D; *n *= 80), respectively. **(E)** HUVECs were treated with or without conditioned medium (CM) from control B16F10 cells or CXCL10-treated B16F10 cells in the presence or absence of VEGF (20 ng/mL). After 4 h, tube formation of HUVECs was observed. Representative images were acquired under an optical microscope (50×) and tube number (/field) was quantified. **(F)** HUVECs were treated with or without CM from control B16F10 cells or CXCL10-treated B16F10 cells in the presence or absence of VEGF (20 ng/mL). After 24 and 48 h, the cells were analyzed by a WST-8 assay. Data are presented as mean ± SD of three independent experiments (**A, E, F**). *, P < 0.05; **, *P *< 0.01; ***, *P *< 0.001, based on the Student’s t-test.

Pro-angiogenic factors, such as VEGF (Rofstad and Danielsen 1998; Rofstad and Halsor 2000), PDGF-B (Barnhill et al. 1996; Rofstad and Halsor 2000), FGF2 (Reed et al. 1994; Miglarese et al. 1997; Rofstad and Halsor 2000), HGF (Lezcano et al. 2014), and ANGPT2 (Helfrich et al. 2009; Abdul Pari et al. 2020), are actively released by human melanoma tumor cells to promote endothelial cell survival, migration, and proliferation, leading to the development of new blood vessels. As CXCL10 was amplified related to the induction of tumor angiogenesis during B16F10 melanoma tumor growth *in vivo*, we further investigated the effect of CXCL10 on expression of pro-angiogenic factors in B16F10 melanoma cells. Quantitative PCR analyses showed that CXCL10 treatment significantly induced mRNA expression levels of *vegf*, *pdgf-b*, *fgf2*, *hgf,* and *angpt2* in B16F10 melanoma cells (Figure 2(A)). To determine the clinical significance of CXCL10-induced expression of pro-angiogenic factors, we performed genomic analyses from TCGA and The Gene Expression Omnibus (GEO) database. *Cxcl10* mRNA expression levels were significantly higher in melanoma tumors than in normal tissues (Figure 2(B)). As expected, levels of *Cxcl10* mRNA expression were positively correlated with expression levels of *vegf*, *pdgf-b*, *fgf2*, *hgf,* and *angpt2* in both cutaneous melanoma (Figure 2(C)) and uveal melanoma (Figure 2(D)).

To further determine whether CXCL10 could enhance tumor-induced angiogenesis *in vitro* as it upregulated expression of pro-angiogenic factors in B16F10 cells, we performed human umbilical vein endothelial cells (HUVECs) tube formation and proliferation assays using conditioned medium (CM) derived from CXCL10-treated B16F10 cells. As shown in Figure 2(E and F), stimulation of CXCL10-treated B16F10 CM significantly enhanced tube formation and proliferation of HUVECs compared with stimulation of untreated B16F10 CM. Taken together, these results indicate that CXCL10 can induce expression of pro-angiogenic factors in B16F10 melanoma cells, which can enhance angiogenesis *in vitro*.

### CXCL10 induces activation of pro-angiogenic and pro-growth signals in B16F10 melanoma cells and directly enhances B16F10 melanoma tumor growth in an *in vitro* 3D culture

**Figure 3.**
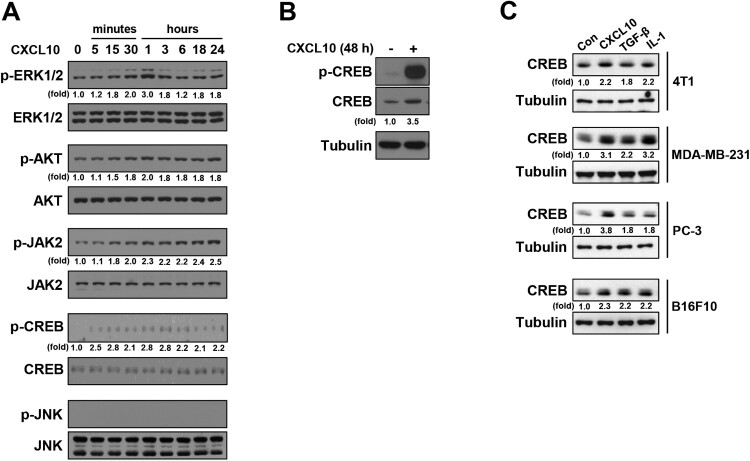
**CXCL10 induces activation of pro-angiogenic and pro-growth signals in B16F10 melanoma cells. (A)** Serum-starved B16F10 cells treated with or without CXCL10 (100 ng/mL) for the indicated period of time. Immunoblotting analyses were performed with indicated antibodies. **(B)** Serum-starved B16F10 cells treated with or without CXCL10 (100 ng/mL) for 48 h. Immunoblotting analyses were performed with indicated antibodies. **(C)** Serum-starved indicated cancer cells treated with or without CXCL10 (100 ng/mL), TGF-β (20 ng/mL) or IL-1 (10 ng/mL) for 48 h. Immunoblotting analyses were performed with indicated antibodies.

We next investigated whether CXCL10 activates signaling pathways involved in the upregulation of pro-angiogenic factors expression, including several MAPK pathways, PI3 K/AKT, JAK/STAT, and CREB (cyclic AMP response element-binding protein) (Deguchi et al. 1999; Wu WZ et al. 2005; Phelps et al. 2006; Jeon et al. 2007; Niu and Carter 2007; Motoki et al. 2008; Pan et al. 2015; Huang et al. 2016; Wu X et al. 2016; Delle Monache et al. 2020; Lu et al. 2022). As shown in Figure 3(A), CXCL10 treatment increased phosphorylation levels of ERK, AKT, and JAK2, but not JNK, in B16F10 cells. In addition, we found that CREB phosphorylation was strongly induced by CXCL10 treatment within 15 min (Figure 3(A)). Interestingly, this phosphorylation was sustained for 48 h accompanied by an upregulation of CREB protein level (Figure 3(B)). CXCL10-upregulated CREB protein levels were also observed in other cancer types, including breast and prostate carcinoma cells (Figure 3(C)), as in transforming growth factor-β (TGF-β)- or interleukin-1 (IL-1)-induced CREB protein upregulation.

**Figure 4.**
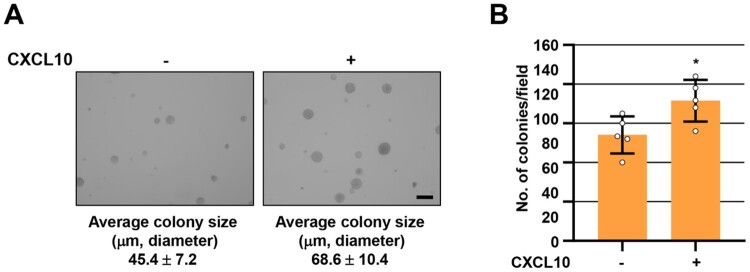
**CXCL10 induces B16F10 melanoma tumor growth in an *in vitro* 3D culture.** B16F10 melanoma cells were cultured on top of a thin layer of Matrigel for 10 days with or without CXCL10 (100 ng/ml). After culturing, colonies were measured (A) and counted (B). Data are presented as mean ± SD of three independent experiments. *, P < 0.05 based on Student’s t-test. Scale bar, 100 μm.

ERK, AKT, and CREB signals, which are activated by CXCL10 (Figure 3), also play important roles in melanoma proliferation and growth (Xie et al. 1997; Savoia et al. 2019; Ma et al. 2020). Therefore, we investigated the effect of CXCL10 on B16F10 tumor growth *in vitro* using an on-top assay involving a basement membrane culture of cells on top of a thin laminin-rich extracellular matrix gel (Lee GY et al. 2007) to mimic the complex three-dimensional (3D) arrangement of tumors *in vivo*. In this condition, B16F10 cells grew as clusters and maintained a 3D structure (Figure 4(A); left) known to play an important role in tumor expansion. Interestingly, CXCL10 treatment significantly enhanced the number of forming colonies compared with untreated control (Figure 4(A and B)). The average colony size was also increased after CXCL10 treatment (68.6 ± 10.4 μm) compared with that in the untreated control (45.4 ± 7.2 μm). Taken together, these results indicate that CXCL10 can activate signal pathways involved in pro-angiogenic and pro-growth in B16F10 melanoma cells and CXCL10 can directly enhance B16F10 tumor growth in an *in vitro* 3D cell culture system.

## Discussion

CXCL10 levels augmented in advanced melanoma patients are associated with poor clinical outcomes (Jiang et al. 2015; Wightman et al. 2015). We and others have previously reported that CXCL10/CXCR3 signaling plays critical roles in melanoma tumor cell motility and metastases to the bone (Lee JH et al. 2012), lung (Wightman et al. 2015), and brain (Doron et al. 2019), accompanied with elevated CXCL10 levels in a mouse model of spontaneous melanoma metastasis. While much data has accumulated on the role of CXCL10 in melanoma metastasis, its role in primary melanoma tumor growth, particularly its host-derived aspects, and the underlying mechanisms remain unclear. In the current report, we showed a significant increase in CXCL10 production during the subcutaneous growth of B16F10 melanoma tumors, primarily originating from the host rather than from the melanoma tumor itself. This melanoma tumor-induced host-expressing CXCL10 played critical roles in melanoma angiogenesis and tumor growth by acting on melanoma cancer cells to induce activation of pro-growth and pro-angiogenic signals and expression of pro-angiogenic factors.

The tumor microenvironment comprises heterogeneous populations, including infiltrating immune cells and stromal cells as well as cancer cells themselves (Binnewies et al. 2018), in which multiple cell types encompassing monocytes, endothelial cells, fibroblasts, inflammatory macrophages and dendritic cells, and cancer cells are responsible for CXCL10 production (Liu M et al. 2011; Tokunaga et al. 2018). Human melanoma cells have been demonstrated to be capable of producing CXCL10 (Harlin et al. 2009), which can be induced by some tumor microenvironmental stimuli, such as IFNγ and toll-like receptor (TLR) agonists (Mauldin et al. 2015). In addition, single-cell RNA sequencing analysis of tumor-infiltrating immune cells in melanoma patients has shown that macrophages are the predominant source of CXCL10 in the melanoma tumor microenvironment (House et al. 2020). Notably, our group has previously demonstrated that melanoma cells can stimulate CXCL10 production from macrophages in a cell-to-cell contact manner and that the augmented production of CXCL10 is required for cancer outgrowth within bone (Lee JH et al. 2012). Results of the current *in vivo* study showed that although intrinsic production of CXCL10 by B16F10 cancer cells contributed to the increase of CXCL10 levels during subcutaneous B16F10 melanoma tumor growth, host-deficiency of CXCL10 almost failed to lead to CXCL10 augmentation (Figure 1(A)) and B16F10 tumor growth (Figure 1(B)), indicating an important role of and the main source of host-derived CXCL10 augmentation induced by interaction between melanoma cancer cells and host-cells or host-microenvironment.

There is increasing evidence that CXCL10 exhibits tumor-promoting abilities in many types of human cancer. Datta *et al*. have reported that Ras-induced overexpression of CXCL10 plays an important role in breast cancer growth (Datta et al. 2006). Interestingly, CXCL10 increases proliferation, migration, and/or epithelial–mesenchymal transition of invasive breast carcinoma cells, hepatocellular carcinoma cells, and lung adenocarcinoma cells through distinct mechanisms, such as upregulation of MMP-1, MMP-2, c-Myc, survivin, β-catenin, and MKP-1 expression or ERK1/2 phosphorylation (Ejaeidi et al. 2015; Ouyang et al. 2016; Duruisseaux et al. 2017; Ren et al. 2017; Kim et al. 2021). CXCL10 can induce glioma proliferation and growth in an ERK1/2-dependent manner (Maru et al. 2008; Liu C et al. 2011). Besides, melanoma tumor clones highly intrinsic expressing CXCL10 can promote melanoma motility, metastasis, and metastatic tumor growth in an autocrine CXCL10/CXCR3 signaling-dependent manner (Wightman et al. 2015) and CXCR3-expressing melanoma cells can promote metastases to the lymph node, bone, and brain in a paracrine-dependent mode of CXCL10 action (Kawada et al. 2004; Lee JH et al. 2012; Doron et al. 2019). In the present study, the amplified CXCL10 mainly from host-cells through interaction with melanoma cells was shown to be critical in melanoma angiogenesis and tumor growth *via* paracrine CXCL10 signaling. Together, these data indicate a wide-range of pro-tumorigenic properties of CXCL10 through both autocrine and paracrine modes of action.

CXCR3 is a G-protein-coupled receptor, and there are three CXCR3 splice variants (CXCR3-A, CXCR3-B and CXCR3-alt) in human cells. These variants have distinct functions, with CXCR3A exerting a pro-tumor effect and CXCR3B playing an anti-tumor role (Reynders et al. 2019). The main variant CXCR3-A found in most cell types associating with the Gαi to activate multiple signaling pathways, including ERK and PI3 K/AKT, thereby inducing cell survival, proliferation and motility (Aksoy et al. 2006; Ji et al. 2008; Maru et al. 2008; Reynders et al. 2019). The dysregulated expression of CXCR3-A in tumor lesions was associated with enhanced tumor development and negative prognosis (Wu Q et al. 2012; Bai et al. 2016; Li H et al. 2019). Indeed, the knockdown of CXCR3-A reduced CXCL10-induced B16F10 cell motility and lymph node metastasis *in vivo* (Kawada et al. 2004). In addition, the proliferative and metastatic potentials conferred by CXCL10-CXCR3-A have been observed in other human cancers, including prostate cancer (Wu Q et al. 2012), gastric cancer (Yang et al. 2016), glioblastoma (Maru et al. 2008), and breast cancer (Kim et al. 2021). In line with previous studies, our current research found that CXCL10 activates pro-angiogenic and pro-growth signals, such as ERK, AKT, JAK2, and CREB, potentially mediated by CXCR3-A, which lead to increased B16F10 tumor growth and angiogenesis.

VEGF is one of the well-known angiogenic factor in regulating angiogenesis and it plays a critical role in continued tumor growth and metastasis (Kieran et al. 2012; Apte et al. 2019). In particular, melanoma overexpresses VEGF levels with highly-developed abnormal vascular structures (Rofstad and Halsor 2000). High levels of VEGF expression in melanoma have been associated with poor prognosis (Cho et al. 2019). Several mechanisms regulating *vegf* gene expression have been studied. Among them, CREB signaling is implicated in VEGF induction (Jeon et al. 2007; Lee JS et al. 2009; Rhee et al. 2015); activated CREB, which is resulted from cAMP/PKA-mediated phosphorylation of CREB at Ser133, directly binds to the VEGF promoter region to induce *vegf* transcription (Jeon et al. 2007). In this study, we found that CXCL10 induces CREB Ser133 phosphorylation in melanoma cells, which could be involved in VEGF upregulation to enhance melanoma angiogenesis.

In conclusion, our *in vitro* and *in vivo* findings showed that CXCL10 expressed by the host in response to melanoma tumor presence exerted pivotal functions in melanoma angiogenesis and vertical tumor growth by stimulating melanoma cancer cells to activate pro-angiogenic and pro-growth signals, which could lead to secretion of distinct pro-angiogenic factors contributing to endothelial cells-mediated angiogenesis and direct melanoma tumor growth. Results presented in the current report support the role of amplified CXCL10 in conferring a pro-tumorigenic function in melanoma development, thereby suggesting a potential strategy to target CXCL10 for melanoma therapy.
